# Exploring the Relationship Between Caring and Missed Nursing Care: A Scoping Review

**DOI:** 10.3390/healthcare14030365

**Published:** 2026-01-31

**Authors:** Gregor Romih, Majda Pajnkihar, Dominika Vrbnjak

**Affiliations:** 1 Fresenius Medical Care, Gaji 28, 3000 Celje, Slovenia; 2 Faculty of Health Sciences, University of Maribor, Žitna ulica 15, 2000 Maribor, Slovenia

**Keywords:** quality of health care, caring, patient safety, nursing care, work environment

## Abstract

**Background/Objectives**: Missed nursing care is a recognized indicator of nursing quality and safety, while caring is a foundational concept in nursing practice. Few studies have empirically examined their relationship. This scoping review aimed to map and synthesize existing evidence on the conceptualisation, measurement approaches, and empirical relationships between caring and missed nursing care. **Methods**: The review was conducted using JBI methodology, reported according to PRISMA-ScR guidelines, and was registered in the Open Science Framework. Literature was searched in PubMed, CINAHL Ultimate (EBSCOhost), MEDLINE (EBSCOhost), and Web of Science, with additional grey literature searches in ProQuest Dissertations & Theses and Google Scholar. The review included studies examining caring in relation to missed nursing care across any healthcare setting. All study designs were considered. Data were extracted using an extraction tool, developed based on JBI guidelines, and piloted. Data were analyzed descriptively, tabulated, and summarized narratively. **Results**: Five quantitative cross-sectional studies met the inclusion criteria, conducted between 2012 and 2024 in the Philippines and Slovenia. Caring was assessed using the Caring Behaviors Inventory, Caring Ability Inventory, or CARE-Q, while missed nursing care was measured using the MISSCARE Survey or the Missed Nursing Care Scale. Most studies used Watson’s Theory of Human Caring, Duffy’s Quality Caring Model, or the Missed Nursing Care Model as theoretical frameworks. Across studies, caring behaviours and caring ability were negatively associated with missed nursing care. **Conclusions**: Caring can function as a moral and relational ideal and as a measurable and actionable factor related to patient outcomes. However, the evidence base remains limited, with inconsistent theoretical foundations and a lack of experimental studies. Future research should adopt theory-based, experimental approaches with diverse samples to explore causal mechanisms and evaluate strategies that strengthen caring competence and caring organizational cultures.

## 1. Introduction

Quality and safety are central to healthcare, aiming to deliver high-quality care that minimizes incidents with potential harm to patients, staff, or others involved in the healthcare process [1]. Errors in healthcare jeopardize patient safety and cause adverse events and compromise overall health quality and safety [2]. Such errors are defined as deviations from the healthcare process that may or may not result in harm [3].

Missed nursing care is an error of omission [4], and a widely monitored indicator of nursing quality and safety [5]. It refers to the partial or complete omission of essential nursing activities necessary for effective patient care (e.g., nourishment, hygiene, ambulation and mobility, medication administration, diagnostic or therapeutic activities, emotional support, teaching, documenting, etc.) [5]. Missed nursing care is understood as a system-level issue arising from resource constraints, organizational factors, and competing care demands, rather than intentional professional omission [6,7,8].

Missed nursing care is a prevalent global problem. A recent systematic review and meta-analysis of 28 studies across 14 countries reported prevalence rates ranging from 6.8% to 98.1%, with a median of 56.4% [9]. European studies report rates ranging from 24 to 62%, commonly involving emotional support, timely responses, patient education, and interdisciplinary communication [10,11]. Contributing factors include nurse-related, patient-related, organizational, and workflow-related factors [9,12,13,14,15,16]. Missed nursing care is associated with adverse patient outcomes, including medication errors, falls, infections, pressure ulcers, and increased mortality [9,17,18].

Caring for the patient is a fundamental value and concept in nursing [19,20,21,22], forming the foundation of professional nursing identity [22]. Caring is conceptualized and operationalized in diverse ways across the nursing literature [23]. Caring is understood as a process of interpersonal interaction encompassing sensitivity, authentic relationships, knowledge, and preservation of human dignity [20,24,25]. It is expressed through attitudes, behaviours, and skills or ability [23,26]. Caring theories such as Watson’s and Duffy’s posit that caring influences care processes and, consequently, patient outcomes [22,26]. Empirical evidence links caring with better patient outcomes, including physical and mental well-being, satisfaction, and quality and safety of care [21,27,28,29,30,31,32,33,34,35,36,37,38,39], and improved nurse outcomes such as psychological well-being and job satisfaction [24,32,37,40,41].

According to Watson’s theory [22], a caring nurse advocates for others’ well-being and safety. By establishing caring interpersonal relationships, nurses influence the sense of being cared for and ultimately affect the care process and outcomes of patients and nurses [42,43]. Duffy’s theory [26] posits that caring leads to positive patient outcomes, particularly in relation to safety. Consequently, missed nursing care is regarded as a quality and safety indicator in nursing [5,44,45].

Although Watson’s theory of human caring [22] and Duffy’s Quality Caring Model [26] link caring relationships to improved care processes and patient safety outcomes, few studies have empirically examined the relationship between caring and missed nursing care. While existing systematic reviews have examined organizational and structural antecedents of missed nursing care [9,12,13,14,15,16], none have specifically investigated caring as a predictor or correlate of missed nursing care. Despite existing knowledge on caring and missed nursing care as separate concepts, no scoping review has, to our knowledge, mapped the empirical literature on caring in relation to missed nursing care. We conducted a preliminary search of PubMed, CINAHL Ultimate (EBSCOhost), MEDLINE (EBSCOhost), Web of Science, the International Prospective Register of Systematic Reviews (PROSPERO), and the Open Science Framework (OSF), which revealed no existing or ongoing systematic or scoping reviews on this topic.

Understanding this relationship may inform interventions that strengthen caring competencies, nursing education, caring-based leadership, and organizational culture to improve patient safety and nurse well-being. Therefore, a scoping review was chosen to systematically map and summarize the limited and heterogeneous evidence examining the relationship between caring and missed nursing care, an underexplored topic despite extensive research on each concept independently.

This scoping review aimed to map and summarize the existing literature on caring in relation to missed nursing care by (1) identifying how caring and missed nursing care are defined and conceptualized in studies examining their relationship; (2) identifying measurement approaches for caring and missed nursing care in these studies; (3) synthesizing empirical findings on the relationship between caring and missed nursing care; and (4) identifying gaps in the current evidence base to inform future research.

This scoping review addresses the following review question: “What is known about the relationship between caring and missed nursing care?”

## 2. Materials and Methods

A scoping review was conducted using the JBI methodology for scoping reviews [46] and reported according to Preferred Reporting Items for Systematic Reviews and Meta-Analysis for Scoping Reviews (PRISMA-ScR) guidelines [47].

The scoping review protocol was registered on the Open Science Framework (OSF) (https://doi.org/10.17605/OSF.IO/PSXU5) on 4 July 2025, to enhance transparency and reduce duplication.

### 2.1. Inclusion Criteria

Inclusion and exclusion criteria were followed for the selection of relevant literature, defined based on the PCC framework (population, concept, context) [46].

Regarding population (P), studies including nurses, regardless of the field of nursing, were included. For concept (C), the review included studies focusing on the conceptualization, measurement, and description of caring in relation to the concept of missed nursing care, including its definitions, measurements, frequencies, and contributing factors. In terms of context (C), the review included studies conducted in any healthcare setting, with no restrictions on country, language, or publication date. All study designs were considered, including experimental, quasi-experimental, analytical, descriptive observational, qualitative, mixed-methods, reviews, and relevant text papers. No restrictions were applied based on language, publication year, or country of origin. If non-English studies requiring translation had been identified, they would have been translated using DeepL version 26 (DeepL SE, Cologne, Germany) with validation by bilingual team members. However, only one non-English study (in Slovenian) was identified and assessed directly by native-speaking authors.

Regarding population (P), studies not including nurses, regardless of nursing field, were excluded. For concept (C), studies not focusing on the conceptualization, measurement, or description of caring in relation to missed nursing care (including its definitions, measurements, frequencies, and contributing factors) were excluded. In terms of context (C), studies conducted outside healthcare settings were excluded, while no restrictions were applied regarding the country or specific type of healthcare setting. Regarding study design, studies not employing experimental, quasi-experimental, analytical, descriptive observational, qualitative, mixed-methods, reviews, or relevant text papers were excluded. Editorials and opinion pieces without empirical data were excluded. No restrictions were applied based on language or publication year. Records for which the full text could not be retrieved were excluded, as were studies whose content did not align with the defined PCC inclusion criteria.

### 2.2. Search Strategy

A literature search was performed in July 2025 in PubMed, CINAHL Ultimate (EBSCOhost), MEDLINE (EBSCOhost), and Web of Science. Additional searches for relevant grey literature were conducted in ProQuest Dissertations & Theses and Google Scholar. Titles, abstracts, and index terms of relevant publications were identified during the preliminary search and used to construct a comprehensive search strategy, which was customized for each database and information source, incorporating all relevant keywords and index terms.

Keywords in English (“missed nursing care”, “caring”) and their synonyms, combined with Boolean operators, were used to formulate the search string. The search strategy was iteratively refined based on preliminary search results. The final search string in all databases and search engines was (caring OR “caring theory” OR “caring behaviour” OR “caring behaviours” OR “caring behavior” OR “caring behaviors” OR “caring attitude” OR “caring attitudes” OR “caring culture” OR “caring management” OR “caring leader” OR “caring leaders” OR “caring environment” OR “caring work environment” OR “caring interventions”) AND (“missed care” OR “care left undone” OR “omitted care” OR “care omission*” OR “unfinished care” OR “incomplete care” OR “care undone” OR “task* undone” OR “task* left undone” OR “rationed care” OR “rationing of care” OR “implicit rationing” OR “implicit rationing* of care” OR “error* of omission”).

In PubMed, CINAHL Ultimate (EBSCOhost), MEDLINE (EBSCOhost), and Web of Science, the search string was applied without field restrictions, searching across all available fields. In Google Scholar and ProQuest Dissertations & Theses, the first 50 results were screened for relevance to balance comprehensiveness with feasibility and included when appropriate.

### 2.3. Source of Evidence Selection

**Figure 1:**
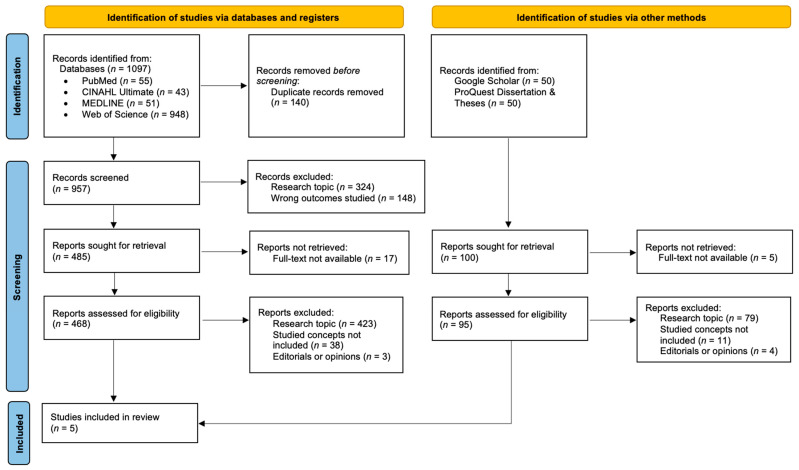
PRISMA flowchart of the study selection process.

The first (GR) (PhD candidate in nursing and teaching assistant) and third (DV) (PhD in nursing and associate professor) authors independently conducted the literature search and assessed the identified records for eligibility. Identified records from databases were imported into EndNote 20 (Clarivate, Philadelphia, PA, USA), where duplicates were removed, followed by title and abstract screening. Full-text screening was performed in Rayyan [48] according to predefined inclusion and exclusion criteria, with reasons for exclusion recorded. The two reviewers then met for three structured consensus meetings (each 60–90 min). The purpose of these meetings was to systematically compare individual screening decisions, review discrepancies in study inclusion or exclusion, re-examine full-text articles against the predefined inclusion criteria, and reach final decisions on inclusion. Consensus was defined as unanimous agreement between the two reviewers (GR and DV) that a study met or did not meet the defined inclusion criteria. When initial assessments differed, both reviewers independently reassessed the study and discussed discrepancies until unanimity was reached. If unanimity could not be achieved after discussion, the second author (MP), a full professor with expertise in caring science, would independently review the study and serve as the third reviewer adjudicator. However, no study required third-reviewer adjudication, as all disagreements were resolved through discussion between the first author and the third author. Additional relevant records were identified in ProQuest Dissertations & Theses and Google Scholar, where the first 50 results were screened. No additional relevant studies were identified in the final 20 records screened, indicating saturation. The study selection process is presented in the PRISMA flowchart (Figure 1) [49].

### 2.4. Data Extraction

Data extraction from the included studies was performed independently by two authors using a structured data extraction form developed based on JBI guidance for scoping reviews [46] and tailored to capture elements relevant to the research objectives. Extracted data included details about aim, research design, setting, sample, sampling method, underlying concepts and theoretical frameworks, concept definitions, data collection methods, and key findings relevant to our research question. The form was piloted on three studies and refined iteratively to ensure consistency and comprehensive data capture. Disagreements that arose between the authors were resolved through discussion.

### 2.5. Data Analysis and Presentation

Data were charted and synthesized using descriptive numerical analysis and narrative summary. Descriptive statistics (frequencies, proportions) were used to summarize study characteristics, populations, settings, and concepts. Formal critical appraisal of methodological quality was not conducted, consistent with the mapping aims of scoping reviews [46]. The results were presented in tabular format, accompanied by a narrative summary.

## 3. Results

### 3.1. Search Results

Initially, 1097 records were identified in the databases. After removing duplicates, 957 records were screened based on their titles and abstracts. Of these, 472 records were excluded based on irrelevant titles and abstracts, leaving 485 records. Of the 485 records, 17 could not be retrieved in full text (despite attempts to access through institutional resources), leaving 468 records for eligibility assessment. In the next step, 423 records were excluded due to irrelevant research topics, not including both studied concepts (caring and missed nursing care), being editorials or opinion pieces. In addition, screening in Google Scholar and ProQuest Dissertation & Theses of 100 records was performed. Of these, five records could not be retrieved in full text (despite attempts to access through institutional resources), leaving 95 records for eligibility assessment. In the next step, 94 records were excluded because they were irrelevant to the research topics, did not include both studied concepts (caring and missed nursing care), or were editorials or opinion pieces. Finally, one record was identified that met the inclusion criteria. A total of five studies were included in the final analysis, as shown in the PRISMA flowchart [49] (Figure 1).

### 3.2. General Characteristics of Included Studies

A summary of the included studies is presented in Table 1, with detailed study characteristics provided in Supplementary Table S1. Four out of five studies were conducted in the Philippines [50,51,52,53] and one in Slovenia [54]. Overall, no relevant studies examining the relationship between caring and missed nursing care were published before 2012. The first study published considering this topic was a master’s thesis in 2012 [54].

### 3.3. Study Aims and Purposes

Overall, the included studies converged on examining how the concepts of caring and missed nursing care are connected in clinical nursing practice. The included studies examined how caring relates to missed nursing care, operationalizing caring as, e.g., caring behaviours or caring ability, across various settings, including emergency, critical care, hospital, and general nursing settings. Labrague and colleagues [51] investigated how nurses’ capacity for compassionate, patient-centred care influences the likelihood of missed nursing care, adverse patient events, and the overall quality of nursing care. Labrague [52] further assessed the caring ability of emergency nurses and its relationship to patient safety outcomes, and to test whether caring ability mediates the link between reality shock and missed nursing care in newly graduated critical care nurses [53]. The study by Berdida and Alhudaib [50] examined the interrelationships between patient safety, caring behaviours, professional self-efficacy, and missed nursing care among emergency room nurses. Močnik [54] aimed to present the concepts of caring and missed nursing care and to determine their relationship in clinical nursing practice. By jointly presenting caring and missed nursing care, these studies aimed to clarify their relationship and highlight their combined impact on patient outcomes in real-world clinical environments.

### 3.4. Study Designs and Data Collection Methods

All included studies were independent quantitative studies, using cross-sectional designs. One study explicitly stated that it used cross-sectional, correlational design.

Data collection was performed via the survey method, using standardized and validated instruments for measuring the concepts of caring and missed nursing care, as other concepts of interest. Across the five studies, five distinct instruments to measure caring and missed nursing care were identified. For measuring missed nursing care, the 12-item Missed Nursing Care Scale by Lake and colleagues [55] was used in four out of five studies [50,51,52,53], and the 41-item MISSCARE Survey by Kalisch and Williams [56] was used in one study [54]. Caring concepts were measured using the 16-item Caring Behaviour Inventory (CBI) [57] in two out of five studies [50,51], the 37-item Caring Ability Inventory (CAI) [58] in two out of five studies [52,53], and the 50-item Caring Assessment Report Evaluation (CARE-Q) [59] in one study [54].

Additional concepts were also measured using the survey method. Five distinct instruments were used. The concept of safety attitudes was measured using the 19–item Safety Attitude Questionnaire (SAQ) [60] in one study [50], nurse professional self-efficacy was measured using the 7-item Nurse Professional Self-Efficacy Scale 2 (NPSES2) [61] in one study [50], perception of adverse patient events was measured using the 5-item Adverse Patient Events (APE) Scale [62] in two out of five studies [51,52], quality of care was measured using single-item scale for nurse-assessed quality of care [63] in two out of five studies [51,52], and the concept of reality shock was measured with the 22-item Environmental Reality Shock-Related Issues (ERS-RIC) scale [64] in one study [53]. All studies included demographic profile questions in their surveys.

**Table 1:** Summary of included studies.

Study	Country and Setting	Design and Sample	Underlying Concept/ Framework	Caring Measurement	Missed Nursing Care Measurement	Key Findings
**[50]**	Philippines, emergency departments	Cross-sectional, *n* = 45 ER nurses	The Missed Nursing Care Model [5]	Caring Behaviour Inventory (CBI) [53]	Missed Nursing Care Scale [51]	Caring behaviours negatively predicted MNC (β = −0.44, *p* < 0.001); patient safety indirectly affected MNC via caring behaviours and self-efficacy
**[51]**	Philippines, 6 hospitals	Cross-sectional, *n* = 549 RNs	Not explicitly stated; implicit reference to Watson’s Theory	Caring Behaviour Inventory (CBI) [53]	Missed Nursing Care Scale [51]	Caring behaviours negatively predicted MNC (β = −0.029, *p* < 0.05) and negatively correlated with MNC (r = −0.106, *p* < 0.05)
**[52]**	Philippines, 10 hospitals	Cross-sectional, *n* = 164 ER nurses	Not explicitly stated or defined	Caring Ability Inventory (CAI) [54]	Missed Nursing Care Scale [51]	Caring ability negatively associated with MNC (β = −0.158, *p* < 0.01)
**[53]**	Philippines, 7 hospitals, critical care	Cross-sectional, *n* = 286 newly graduated nurses	Conservation of Resources Theory [61]	Caring Ability Inventory (CAI) [54]	Missed Nursing Care Scale [51]	Caring ability negatively associated with MNC (r = −0.106, *p* < 0.05) and mediated reality shock-MNC relationship
**[54]**	Slovenia, clinical nursing settings	*Cross-sectional*, *n* = 83 nurses	Watson’s Theory of Human Caring, Kalisch concept of missed nursing care	Caring Assessment Report Evaluation (CARE-Q) [55]	MISSCARE Survey [52]	Caring negatively correlated with MNC (r = 0.415, *p* < 0.05); all CARE-Q dimensions correlated with MNC

*n*, sample size; ER, emergency room; RN, registered nurse; MNC, missed nursing care; *p*, *p* value; significance, r; correlation coefficient, β, standard regression beta coefficient.

### 3.5. Study Sample and Settings

Four out of five studies reported the response rate [50,51,52,53], whereas one study did not report it [54]. In total, out of 1700 invited nurses, 1344 nurses participated in these four studies (79.1% response rate). Response rates from these four individual studies ranged from 60.2% [50] to 95.3% [53]. Cumulatively, 1427 nurses (including the study by Močnik [54]) participated, with the highest number being reported in the study by Labrague and colleagues [51] (*n* = 549 nurses) and the lowest in the study by Močnik [54] (*n* = 83). Four out of five studies used convenience sampling [50,51,52,53], while one study used snowball sampling via an online survey (FluidSurveys Ultra) [54]. The study setting in two out of five studies involved an emergency nursing setting and included emergency nurses [50,52], one involved a critical nursing setting [53], one involved a clinical nursing practice setting [54], and one did not specify the nursing setting, just Registered Nurses working in a hospital [51]. Data in the included studies took from three to five months to collect.

### 3.6. Studies’ Theoretical Frameworks and Definitions of Concepts

Across the included studies, theoretical frameworks for examining missed nursing care, caring ability, and caring behaviours were variably described. The Missed Nursing Care Model by Kalisch [5] was referenced in two out of five studies [50,54], which used it to support empirical models linking patient safety, caring behaviours, professional self-efficacy, and missed nursing care. Močnik [54] used the Kalisch missed nursing care concept definition. Two out of five studies [51,54] drew upon Watson’s Theory of Human Caring [22] and Duffy’s [26] perspective, emphasizing that stronger nurse caring behaviours may reduce missed nursing care and adverse events, thereby improving care quality. The Conservation of Resources Theory [65] was also applied in one study [53], conceptualizing caring ability as a personal resource that mediates care delivery. This framework suggests that stressors such as reality shock may deplete emotional resilience, empathy, and communication skills, which in turn reduce caring ability and increase the incidence of missed nursing care. In the study by Labrague [52], no theoretical foundations or backgrounds were defined.

Concept definitions for missed nursing care were inconsistent across studies, though defined in four out of five studies [50,52,53,54]. Definitions generally aligned on the omission or delay of essential patient care activities that could threaten patient safety. Descriptions ranged from broad definitions encompassing any required nursing care omitted or delayed, to more specific formulations highlighting partial or complete omissions and delays in care delivery [6,7,66].

The concept of caring was explicitly defined in one study [54], and the definition was related to Watson’s Theory of Human Caring [22] as a fundamental concept in nursing that includes the nurse’s attitude and relationship with patients. Caring behaviours were not explicitly defined in two out of five studies [50,51] but were commonly described in terms of nurse actions that demonstrate concern, empathy, compassion, and professional engagement. Caring ability on the other hand, was used in two out of five studies [52,53], but explicitly defined in one [52]. The definition emphasized a nurse’s capacity and skill to provide compassionate, empathetic, and patient-centred care to individuals under their supervision [67].

### 3.7. Relationship of Caring with Missed Nursing Care

Across all five studies, a consistent negative relationship emerged between caring and missed nursing care. Studies examining caring behaviours [50,51,54] demonstrated that nurses who exhibited higher levels of caring behaviours reported lower frequencies of missed nursing care. Similarly, studies focusing on caring ability [52,53] found that nurses with greater caring ability were less likely to omit or delay essential care activities.

The strength of these associations varied from weak to moderate, with caring explaining between 1.2% and 17.6% of the variance in missed nursing care. Notably, caring ability also functioned as a partial mediator in the relationship between reality shock and missed nursing care [53], suggesting that caring serves as a protective resource that helps sustain nursing quality even under adverse working conditions.

These findings were consistent across different operationalizations of caring (behaviours, ability, perceived caring) and different healthcare settings (emergency departments, critical care, general hospital nursing).

### 3.8. Barriers and Facilitators

Missed nursing care was more common when nurses reported lower patient safety perceptions, weaker caring behaviours, and low self-efficacy [50]. Additional barriers included working in large hospitals, which was linked to reduced caring ability [52], and experiencing reality shock, which significantly increased missed care [53]. In some studies, barriers were not explicitly reported [51,54].

On the other hand, facilitators consistently centred on strong caring behaviours and abilities. High levels of caring behaviours and professional self-efficacy were shown to reduce missed care and buffer the negative effects of poor safety perceptions [50] and reality shock [53]. Nurses with stronger caring ability also reported higher perceived quality of care, fewer adverse patient events, and lower missed nursing care [51]. Furthermore, caring behaviours were associated with lower perceptions of missed nursing care overall [52,54], reinforcing the protective role of caring attitudes and skills in clinical practice.

### 3.9. Other Key Findings

Beyond the direct caring and missed nursing care relationship, several additional patterns emerged across studies. Caring behaviours and professional self-efficacy functioned as mediators between organizational factors (patient safety climate, reality shock) and missed nursing care [50,53].

Across the Philippine studies [50,51,52,53], nurses generally reported moderate to high levels of caring alongside relatively low frequencies of missed nursing care. The Slovenian study [54] provided detailed data on specific caring dimensions, with monitoring rated highest and anticipating rated lowest among caring behaviours. For missed nursing care, blood glucose monitoring was least frequently missed, while patient ambulation was most frequently missed.

Three Philippine studies [50,52,53] reported only overall missed nursing care scores, while one [51] identified specific frequently missed activities including comforting patients, repositioning, skincare, oral hygiene, and care plan development.

## 4. Discussion

This scoping review identified five studies examining the relationship between caring and missed nursing care. A consistent negative relationship emerged, with caring behaviours and caring ability associated with lower frequencies of missed nursing care across diverse healthcare settings. However, the evidence base is limited, geographically concentrated, and characterized by substantial theoretical and methodological heterogeneity. The authors of included studies recommend establishing supportive work environments with adequate staffing, strengthening nurses’ caring abilities through training and mentorship, and expanding research using robust longitudinal designs to explore causal relationships between caring behaviours and patient outcomes.

The included studies operationalized and examined caring primarily through two dimensions: caring behaviours [50,51,54] and caring ability [52,53]. Caring behaviours were commonly described in terms of nurse actions that demonstrate concern, empathy, compassion, and professional engagement [50,51], though not all studies provided explicit definitions. Caring ability was defined as a nurse’s capacity and skill to provide compassionate, empathetic, and patient-centred care to individuals under their supervision [67]. Only one study explicitly defined the concept of caring, relating it to Watson’s Theory of Human Caring as a fundamental concept in nursing that includes the nurse’s attitude and relationship with patients [54]. This represents a notable conceptual gap in the literature, as caring was more frequently operationalized and measured than formally defined.

Definitions of missed nursing care were more consistent across studies, with four out of five studies providing explicit definitions [50,52,53,54]. These generally aligned with Kalisch’s conceptualization on the omission or delay of essential patient care activities that could threaten patient safety, ranging from broad definitions encompassing any required nursing care omitted or delayed, to more specific formulations highlighting partial or complete omissions and delays in care delivery [6,7,66].

Substantial theoretical inconsistency emerged across studies. Studies included in the analysis used prominent nursing theories as theoretical frameworks, such as Kalisch’s Missed Nursing Care Model [5], Watson’s Theory of Human Caring [22], Duffy’s Quality-Caring Model [26], and the Conservation of Resources Theory [65], to define and explain their hypothesized conceptual models, but only three of five studies explicitly identified a theoretical framework, with one providing no theoretical foundation at all [52].

More critically, measurement instruments were often not derived from the stated theoretical frameworks, creating misalignment between conceptual foundations and empirical operationalization. Lake and colleague’s [55] Missed Nursing Care Scale was developed based on empirical research findings [68,69,70,71,72]. Specifically, the authors of the scale did not explicitly state the distinct theoretical framework on which they developed it. Kalisch and Williams’s [56] MISSCARE Survey, however, is derived from Kalisch’s model and her conceptualization of missed nursing care, although this was explicitly referenced only in the study by Močnik [54]. Despite the general use of Kalisch’s conceptual framework to define the missed nursing care concept, the measurement instruments employed in the studies were not fully consistent with the established operationalizations of the construct. Furthermore, for the caring perspective, both Wolf and colleagues’ Caring Behaviour Inventory (CBI) [57] and Larson’s [59] Caring Assessment Report Evaluation Questionnaire (CARE-Q) are derived from Watson’s Theory of Human Caring, which was utilized in some of the studies. On the other hand, Nkongho’s [58] Caring Ability Inventory (CAI) is grounded in Mayeroff’s definition of caring [73], which was likewise not applied in the included studies.

The relationship between caring behaviours or caring ability with missed nursing care was consistently negative. Correlations ranged from weak to moderate (r = −0.11 to −0.42), explaining 1.2–17.6% of variance [50,51,52,53,54]. Higher caring behaviours or ability predicted lower frequencies of missed care [50,51,52,53]. Although effect sizes were modest, these findings were consistent across studies, suggesting a stable relationship. Additionally, caring ability partially mediated the relationship between reality shock and missed nursing care, suggesting that caring competence serves as a protective resource sustaining nursing quality under challenging conditions [53].

These findings collectively emphasize the critical role of caring as a fundamental concept of professional nursing practice. In clinical practice, higher levels of nurses’ caring behaviours and caring ability are associated with fewer missed nursing care activities, or vice versa. This relationship underscores that caring not only reflects professional values but also enhances situational awareness, communication, and patient engagement, thereby reducing the likelihood of missed nursing care. The negative association between caring and missed nursing care suggests that nurses who possess greater empathy, compassion, and interpersonal sensitivity are more likely to prioritize and complete essential nursing activities. By fostering caring, empathetic, and compassionate care, healthcare institutions may effectively mitigate the occurrence of missed nursing care, thereby enhancing both the quality and safety of patient outcomes.

This aligns with theoretical premises that caring relationships positively influence patient safety and care quality [22,26]. A caring organizational culture, supported by leadership, serves as both a prerequisite and foundation for safe, high-quality nursing care [38,74,75].

Several important gaps were identified. Despite a comprehensive search, only five studies explicitly examined this relationship, highlighting notable research scarcity. All employed cross-sectional survey designs with convenience-based sampling, limiting generalizability and precluding causal inference regarding the directionality of the caring and missed nursing care relationship.

Second, there was substantial geographical bias, with four of five studies conducted exclusively in the Philippines and only one in Slovenia. This highlights the lack of evidence from diverse health systems, organizational cultures, and socio-economic contexts. Geographical narrowness constrains the global understanding of how caring manifests and impacts nursing outcomes in various settings. This geographical homogeneity represents a critical gap in the evidence base and indicates an urgent need for research from diverse international contexts, particularly from high-income Western, Middle Eastern, African, and Asian healthcare systems beyond the Philippines.

Third, conceptual ambiguity was identified regarding the operationalization of caring. Studies examined caring through caring behaviours [50,51,54], caring ability [52,53], and perceived caring [54], representing different dimensions (actions, competence, perceptions) that are not directly comparable. Moreover, caring was explicitly defined in only one study [54]. Additionally, patterns of missed nursing care varied across studies. Methodological heterogeneity, including inconsistent reporting of specific missed nursing activities and use of different measurement scales, limits direct comparison of these patterns. Sample sizes also varied considerably, ranging from 83 to 549 participants, raising concerns about statistical power in smaller studies. This heterogeneity, combined with theoretical inconsistencies, severely limits the comparability of findings about the caring and missed care relationship.

Despite the identified gaps, findings across the included studies demonstrate that caring behaviours and caring ability are forms of protective factors against missed nursing care. Nurses who demonstrated higher empathy, compassion, and interpersonal sensitivity reported lower frequencies of missed nursing care and perceived a higher quality and safety of patient care. The results support the theoretical premise that caring is not only a moral and professional foundation but also a central concept influencing patient safety and quality of care [38]. Embedding caring competencies in undergraduate nursing education and leadership training may strengthen professional values and reduce care omissions. These findings highlight the importance of fostering caring-based organizational cultures as a strategic priority for improving nursing quality and patient safety.

Future research should explore how leadership and organizational factors may moderate the relationship between caring and missed nursing care. Longitudinal and interventional research is needed to establish causal relationships and test interventions aimed at strengthening caring competencies. Future studies should employ diverse, representative samples from multiple countries and healthcare contexts, use theory-driven instruments, and apply explicit theoretical frameworks that clearly define and operationalize caring.

This scoping review has several limitations that should be acknowledged when interpreting the findings. The literature search was conducted in July 2025 across PubMed, CINAHL Ultimate, MEDLINE, Web of Science, in addition to ProQuest Dissertations & Theses and Google Scholar. For grey literature sources (ProQuest Dissertations & Theses and Google Scholar), screening was limited to the first 50 results, which may have excluded potentially relevant studies. Relevant studies in other databases not searched or published after this date may exist. As is typical for scoping reviews, the methodological quality of the included studies was not formally appraised, and therefore, the robustness and credibility of individual findings cannot be fully ascertained. Additionally, the possibility of publication bias cannot be ruled out. Data extraction and charting processes, though guided by established frameworks, involve an element of subjectivity that could influence the classification and interpretation of findings. Furthermore, variations in definitions and conceptualizations of key constructs across studies may have affected the synthesis and interpretation of results. Finally, given the descriptive nature of the scoping review methodology, the findings should be viewed as an overview of existing evidence rather than as a definitive assessment of effectiveness or causality.

## 5. Conclusions

This scoping review reveals a negative association between caring and missed nursing care, indicating that higher levels of caring behaviours and caring ability are associated with lower frequencies of missed nursing care. These findings suggest that caring is not only a professional value but also a valuable resource for enhancing nursing quality and patient safety. For nursing practice, strengthening caring competencies may help reduce missed nursing care by fostering supportive work environments, ensuring adequate staffing and effective teamwork, and providing targeted education, mentorship, and reflective practices. For nursing management and policy, findings highlight the need to integrate caring and missed nursing care as measurable quality indicators, address structural constraints such as workload and resource limitations that undermine caring practice, and foster caring cultures and caring leadership through caring-focused leadership development programs. For research, the current evidence base remains limited by cross-sectional designs and geographic concentration; therefore, longitudinal and intervention-based studies across diverse healthcare settings are needed to establish causal pathways and to evaluate whether caring-focused organizational and educational interventions can sustainably reduce missed nursing care and improve patient and nurse outcomes.

## Data Availability

No new data were created or analyzed in this study.

## Supplementary Materials

The following supporting information can be downloaded at: https://www.mdpi.com/article/10.3390/healthcare14030365/s1. Table S1. Analysis of included studies.

## Guidelines and Standards Statement

This manuscript was drafted against the Preferred Reporting Items for Systematic reviews and Meta-Analyses extension for Scoping Reviews (PRISMA-ScR) [43].
