# Supplementary material for: Exploring the Relationship Between Caring and Missed Nursing Care: A Scoping Review
Source: Healthcare (Basel). 2026 Jan 31;14(3):365. doi: 10.3390/healthcare14030365 (PMC12896982; doi:10.3390/healthcare14030365)
Supplement: Supplementary file 1 [file healthcare-14-00365-s001.zip › healthcare-4016742-supplementary.pdf]

Table S1. Analysis of included studies.

Source	Aim	Design	Sample	Underlying concept/ framework	Concept definitions	Data collection method/ instruments	Outcomes	Barriers/ facilitators	Other key findings	Recommendations
[50]	Investigate relationships between patient safety, caring behaviours, professional self-efficacy, and MNC among emergency room nurses.	Cross-sectional, correlational study	**Sample:** 345 emergency room Philippino nurses (response rate 60.17%) **Sampling method:** Convenience sampling **Sampling date:** September 2023 – January 2024	The Missed Nursing Care Model [5].	**MNC:** The incapacity or delay in providing essential patient care that potentially threatens patient safety [62]. **CB:** not specifically defined; description related to nurse actions reflecting concern, empathy, and professional engagement.	**Survey method:** Safety Attitude Questionnaire (SAQ) [56]; Caring Behaviour Inventory (CBI) [53]; Missed Nursing Care Scale [51]; Nurse Professional Self-Efficacy Scale 2 (NPSES2) [57].	Patient safety had positive effects on (p z .05): CB's (β = 0.47), professional self-efficacy (β = 0.37), MNC (indirect effect via mediators: β = –0.31). CB's negatively predicted MNC (β = –0.44). Professional self-efficacy negatively predicted MNC (β = –0.22, $p < .001$).	**Barriers:** lower patient safety perception, weaker CB's, and low self-efficacy associated with higher MNC. **Facilitators:** high levels of CB's and professional self-efficacy reduced MNC and mediated the negative effects of poor safety perceptions.	The mean of CB's was 4.23 (SD = 0.67) and of the MNC were 1.98 (SD = 0.49) (clinical), and 1.58 (SD = 0.49) (planning and communication). CB's and professional self-efficacy served as mediators between patient safety and MNC. SEM model indicated a strong combined influence of the predictors.	Maintaining a workplace culture patient safety, CB's and professional self-efficacy to minimize avoidable errors, injuries and MNC. Further research for exploring the casualty determination of CB to MNC (more robust study designs). Employment of variables of the role of workplace/ organizational culture, nurse managers' and hospital administrators' role in patient safety.

Source	Aim	Design	Sample	Underlying concept/ framework	Concept definitions	Data collection method/ instruments	Outcomes	Barriers/ facilitators	Other key findings	Recommendations
[51]	Examine the predictive role of nurse CB's on MNC, APE's and the quality of nursing care.	Cross-sectional study	**Sample:** 549 RN's from 6 hospitals in the Philippines (response rate 91.5%) **Sampling method:** Convenience sampling **Sampling date:** October 2018 – January 2019	Not explicitly stated or defined. Appears to be guided by prior research and Watson's theory. Study may also incorporate Duffy's perspective.	**MNC:** not specifically defined. **CB:** not specifically defined.	**Survey method:** Caring Behaviour Inventory (CBI) [53]; Missed Nursing Care Scale [51]; Adverse Patient Events (APE) Scale [58]; Single-item scale for nurse-assessed quality of care [59].	CB's negatively related ($p < .05$) with MNC ($r = -.106$) and APE ($r = -.148$), and positively with PQoC ($r = .246$). APE negatively related ($p < .05$) with PQoC ($r = -.129$). CB's positively predicted PQoC ($\beta = 0.199$, $p \leq .001$) and negatively predicted ($p < .05$) MNC ($\beta = -0.029$) and APE ($\beta = -0.203$).	**Barriers:** not specifically tested or reported in the results. No testing or comparing the measured concepts with demographics reported. **Facilitators:** higher CB's predicted reduced MNC and fewer APE.	CB's were above the midpoint ($M = 4.21$, $SD = 0.66$); PQoC was high ($M = 3.03$, $SD = 0.67$). MNC was perceived as low ($M = 1.21$, $SD = 0.17$), as were the APE ($M = 1.18$, $SD = 1.09$).	Developing a workplace policy to monitor MNC and errors, fostering a positive and ethical workplace culture, ensuring adequate resources and staffing, integrating CB's into professional development and building a caring organizational culture is enhanced. Future research should expand its focus to the associations with other outcomes, exploring the causal relationships (using robust tools and a representative sample), and exploring the effectiveness of interventions.

Source	Aim	Design	Sample	Underlying concept/ framework	Concept definitions	Data collection method/ instruments	Outcomes	Barriers/ facilitators	Other key findings	Recommendations
[52]	Investigate the extent of nurse CA among emergency nurses and its association with patient safety outcomes.	Cross-sectional study	**Sample:** 164 emergency nurses from 10 hospitals in the Philippines (response rate 82%) **Sampling methid:** Convenience sampling **Sampling date:** January 2022 – March 2022	Not explicitly stated or defined.	**MNC:** any aspect of required nursing care omitted or delayed [66]. **CA:** a nurse's capacity and skill to provide compassionate, empathetic, and patient-centered care to individuals under their supervision [65].	**Survey method:** Caring Ability Inventory (CAI) [54]; Missed Nursing Care Scale [51]; Adverse Patient Events (APE) Scale [58]; Single-item scale for nurse-assessed quality of care [59].	CA was positively associated with higher care quality (β = .259, $p < .001$), and lower APE (β = –.169, $p < .05$), and MNC (β = –.158, $p < .01$).	**Barriers:** working in large hospitals was associated with lower CA. **Facilitators:** higher CA was significantly associated with higher perceived quality of care, fewer APE and less MNC.	Mean CA was 67.89/80, indicating moderate nurse CA. PQoC was high (M = 4.83), while MNC (M = 1.20) and APE (M = 1.03) were low. CA correlated ($p < .05$) with PQoC (r = .209) and APE (r = .179). PQoC negatively correlated with MNC (r = -.159).	Promote and enhance nurse CA through training programs. Create supportive and positive work environment with fostering teamwork, communication and recognition systems. Adress workload and staffing with considering the benefits of part-time work models. Implement standardized safety protocols. Future research should explore contextual and organizational factors, explore the causal relationships, and investigate interventions that enhance CA.

Source	Aim	Design	Sample	Underlying concept/ framework	Concept definitions	Data collection method/ instruments	Outcomes	Barriers/ facilitators	Other key findings	Recommendations
[53]	Examine whether CA mediates the relationship between reality shock and MNC among newly graduated critical care nurses.	Cross-sectional study	**Sample:** 286 newly graduated nurses (< 2 years' work experience) from 7 hospitals in the Philippines (response rate 95.3%) **Sampling method:** Convenience sampling **Sampling date:** March 2022 – July 2022	Conservation of Resources Theory [61].	**MNC:** Failure to complete essential patient care activities or the delay in delivering them [63]. **CA:** not specifically defined	**Survey method:** Missed Nursing Care Scale [51]; Environmental Reality Shock-Related Issues (ERS-RIC) scale [60]; Caring Ability Inventory (CAI) [54]	Reality shock positively associated with MNC (β = .0493, p = .007); CA partially mediated this relationship (β = .004, SE = .003).	**Barriers:** reality shock significantly predicted higher levels of MNC. **Facilitator:** higher CA reduced the negative impact of reality shock on MNC.	Reality shock was moderate (M = 50.31, SD = 1.17). CA was high (M = 221.03, SD = 20.8). MNC was low (M = 13.44, SD = 2.01). Reality shock positively correlated with MNC (r = .116, p < .01). CA negatively associated (p < .05) with both MNC (r = -.106) and reality shock (r = -.102).	Support transition with mentorship and structured onboarding. Promote a positive work environment (communication, psychological safety, teamwork, adequate staffing). Enhance caring competence through training and performance evaluation. Integrate caring models and encourage continuous professional development. Future research should use rigorous methods, examine mediators, and test interventions to improve CA.

Source	Aim	Design	Sample	Underlying concept/ framework	Concept definitions	Data collection method/ instruments	Outcomes	Barriers/ facilitators	Other key findings	Recommendations
[54]	Present the concepts of caring and MNC and to determine their relationship in clinical nursing practice.	Cross-sectional study	**Sample:** 83 nurses from clinical nursing practice settings in Slovenia **Sampling method:** Snowball sampling via online survey (FluidSurveys Ultra). **Sampling date:** March 2012 – June 2012	Watson's Theory of Human Carin. Kalisch concept of missed nursing care.	**MNC:** Omission or delay of required nursing activities, partially or in whole [64]. **Caring** as fundamental concept in nursing that includes the nurse's attitude and relationship with patients conceptualized through Watson's theory.	**Survey method:** Caring Assessment Report Evaluation (CARE-Q) [55]; MISSCARE Survey [52].	Perceived caring negatively correlated ($p < .05$) with MNC using reverse scoring ($r = .415$). CARE-Q dimensions Consolation and comforting ($r = .377$), Anticipations ($r = .295$), Trust ($r = .270$), Monitoring ($r = .358$), Accessibility ($r = .315$, $p < .005$) also positively (reverse scoring) correlated with MNC.	**Barriers:** Not specifically reported. **Facilitators:** higher caring associated with lower perceptions of MNC.	Monitoring was the highest perceived dimension of CARE-Q ($M = 6.34$, $SD = .52$) while Anticipating was the lowest ($M = 5.18$, $SD = .98$). The lowest rated MNC item was the one related to blood glucose monitoring ($M = 4.8$, $SD = .62$) and the highest related to patient ambulation ($M = 3.8$, $SD = 1.25$).	Nursing managers should address systemic factors contributing to MNC. Ongoing education should emphasize caring as a core nursing competency.

APE, adverse patient events

CA, caring ability

CB, caring behaviours

M, average

MNC, missed nursing care

n, sample size

p, p value, significance

PQoC, perceived quality of care

r, correlation coefficient

RN, registered nurse

SD, standard deviation

SE, standard error

β, standard regression beta coefficient
